# The Grand Challenge of Characterizing Ribonucleoprotein Networks

**DOI:** 10.3389/fmolb.2016.00024

**Published:** 2016-06-09

**Authors:** Gian Gaetano Tartaglia

**Affiliations:** ^1^Bioinformatics and Genomics, Gene Function and Evolution, Bioinformatics and Genomics Programme, Centre for Genomic Regulation, The Barcelona Institute of Science and TechnologyBarcelona, Spain; ^2^Universitat Pompeu FabraBarcelona, Spain; ^3^Institucio Catalana de Recerca i Estudis AvançatsBarcelona, Spain

**Keywords:** ribonucleoprotein networks, protein–RNA granules, non-coding RNA, high-throughput screening assays, molecular evolution

Protein–RNA interactions are at the heart of cell regulation. From transcription, processing, storage, and translation, all the stages in the life cycle of an RNA depend on interactions with proteins. Although technologies are making remarkable progress in unraveling the landscape of protein–RNA interactions, many key issues are unclear. We still have to identify how many proteins have RNA-binding ability, what are their targets and functional pathways. Moreover, while we know the number of protein-coding genes in the human genome, functional non-coding RNAs are still poorly defined. What is the function of the non-coding part of the eukaryotic transcriptome? A clear understanding of the biological functions of coding and non-coding transcripts would provide novel insights in molecular biology. What are the protein components binding to an RNA while it is being produced? Our lack of understanding of how ribonucleoprotein complexes assemble is a major rate-limiting factor to future progress in the field. We need to generate an in-depth characterization of protein–RNA complexes that form in cells during development and in response to external *stimuli*.

## How did life begin and what role did RNA and protein molecules play?

Life on earth might descend from an RNA world (Higgs and Lehman, 2015) although RNA and proteins could have emerged together. As protein-based molecules are essential to make nucleic acid polymers and nucleotide-based molecules are needed to synthesize proteins, protein, and RNA might have co-evolved from the very beginning of life (Chao et al., 2008): RNA would contain the instructions for life while peptides accelerate key chemical reactions to carry out the instructions. In support of this hypothesis, it has been reported that network of reactions beginning with hydrogen cyanide and hydrogen sulfide in streams of water irradiated by UV light could produce the chemical components of proteins and lipids, alongside those of RNA (Patel et al., 2015).

## Different types of protein-RNA assemblies

From birth to degradation, most cellular RNAs are never naked but they form complexes with partner proteins in ribonucleoprotein (RNP) particles. The assembly of functional RNPs and delivery to their destinations often involves progression through a series of intermediate complexes and subcellular compartments. For instance, *Cajal* bodies are sites of non-coding RNA maturation, concentrating assembly factors to accelerate complicated biochemical reactions (Gall, 2000). Similarly, messenger RNA bind to protein complexes that undergo constant remodeling as they travel from the site of transcription to the cytoplasm (Matera and Wang, 2014). What are the protein components binding to coding and non-coding RNAs while they move in the cell?

Many protein–RNA assemblies are observed in the nucleoplasm and cytoplasm of all cells and play fundamental roles in growth, development, and homeostasis. For instance, using an electron microscope, George Emil Palade observed dense particles or granules in the endoplamic reticulum, which were later called ribosomes (Palade, 1955). Before their export to the cytoplasm, ribosome components are subject to a number of reactions in the *nucleoli*. Processing of the ribosomal RNAs initiates in the dense fibrillar part of the nucleolus and continues in the granular component, where the RNAs self-assemble with ribosomal proteins to form nearly completed subunits. Other examples of protein–RNA assemblies found in the nucleoplasm include speckles and promyelocytic leukemia bodies, among others (Spector and Lamond, 2011). Changes in the protein or RNA composition of these structures are associated with diseases such as Huntington's and spinal muscular dystrophy (Irimia et al., 2014).

**Figure 1:**
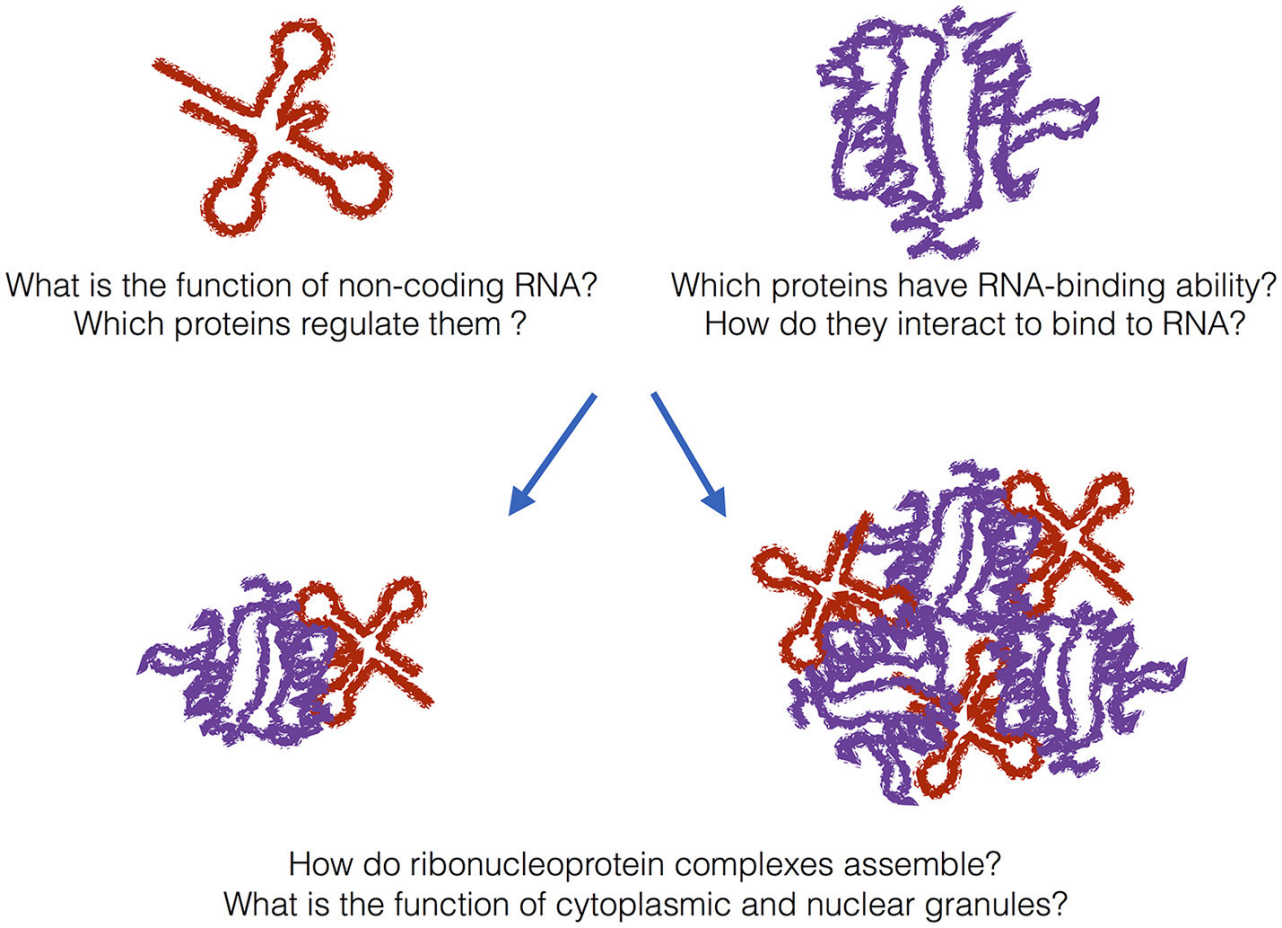
**The grand challenge**. In addition to messenger RNAs, many non-coding transcripts have been found in eukaryotic cells. What is their function and which proteins regulate them? A previously unknown number of proteins bind to RNA even if they do not harbor canonical RNA-binding domains. How do metabolism and transcriptome interact with each other? In addition to canonical ribonucleoprotein complexes, granular assemblies have been found in the cytoplasm and nucleus. What is their biological function and are they also associated with disease?

Studies on the composition, structure, and behavior of speckles provide precious information to understand how protein and RNA components assemble and re-organize in RNP particles such as the spliceosome (Papasaikas et al., 2015). Although highly dynamic, ribosomal, and spliceosomal components form distinct RNPs that involve hundreds of proteins and RNAs (Figure 1): How variable are protein–RNA assemblies in composition? How large, dynamic and structurally heterogeneous is the *spectrum* or ribonucleoprotein assemblies?

## What is the composition of protein-RNA granules inside the cell?

As shown by Tony Hyman and coworkers, RNP self-assemble from soluble proteins and RNAs to form structures that grow, collapse, and fuse continuously (Brangwynne et al., 2009). These and other findings have challenged the concept of cell organization in compartments and how we study protein accumulations (Li et al., 2012). For instance, it has been shown that membrane-less granules form upon environmental insults to prevent translation and when the stress is resolved, the assembly dissolve and protein production is resumed (Buchan and Parker, 2009). Intriguingly, if the stress persists, RNA within the granules can be transferred to other RNP assemblies, called P-bodies, to be degraded (Parker and Sheth, 2007). Recent evidence suggests that ribonucleoprotein granules are also associated with the onset of neurodegenerative diseases (Wolozin, 2012).

We need to generate an in-depth characterization of protein–RNA granules that form in cells during development and in response external *stimuli*. These RNA structures assemblies play key roles in numerous aspects of cell biology and a better understanding of how and why they form will provide significant innovation (Figure 1). We do not have a full biochemical composition for many of these particles: What is the full range and relevance of RNA structures forming granules? Are non-coding RNA participating in RNP assemblies?

## What is the function of the long non-coding part of the transcriptome?

The human genome project was completed in the early 2000s (Lander et al., 2001). Recently, impacts of next-generation sequencing technologies have been massive. Plans to sequence 1000 different human genomes have been pursued and completed. Thousands of different bacteria species have been sequenced, and now over a hundred different plant species have been sequenced. Thanks to Roderic Guigo' and other contributors of the ENCODE project, we now know that a large portion of the genome is transcribed into RNA, but not translated into proteins, resulting in more than half a million of non-coding RNA in eukaryotes (Djebali et al., 2012). Are these non-coding RNA species some kind of transcriptional noise or do they have a biological function? On the one hand, since no gene promoter can be considered silent at all times, ultra-deep sequencing could just reveal single copy RNA transcripts with little functional significance. On the other hand, non-coding RNA could be regulating a number of coding-genes contributing to the complexity of higher eukaryotes. A clear understanding of the biological functions of non-coding transcripts will provide novel insights into our understanding of the molecular biology (Figure 1). What protein networks assist the long non-coding RNAs and what is their specificity?

## How many proteins have RNA-binding ability?

Deep-sequencing approaches based on pull-downs of proteins (e.g., PAR-CLIP and iCLIP) and RNAs (e.g., CHART and CHIRP) as well as *in vitro* evolution methods (e.g., SELEX) have started to unveil the targets of a number of RNA-binding proteins in the cell at defined conditions (König et al., 2010; Bechara et al., 2013; Chu et al., 2015; Darmostuk et al., 2015). Despite the growing amount of data collected, many questions remain to be answered. As shown by the groups of Mattias Hentze and Markus Landthaler, a previously unknown number of proteins have RNA-binding ability, although they do not harbor canonical RNA-binding domains (Baltz et al., 2012; Castello et al., 2012). Intriguingly, a large fraction of these proteins, such as for instance iron responsive protein IRP-1 (Philpott et al., 1994, 1; Cirillo et al., 2013), have a parallel, or moonlighting, activity as metabolic enzymes (Beckmann et al., 2015) (Figure 1). How do transcriptomic and metabolomic pathways interact with each other in the cell? What are the targets of the newly discovered RNA-binding proteins?

## Let the grand challenge begin!

The field of protein–RNA interactions is moving fast and a number of fascinating hypotheses have been recently formulated on ribonucleoprotein complexes. A PubMed search using the terms “protein” and “RNA” shows that the number of indexed publications increased progressively in the last decades: 736 (period 1985–1995), 1377 (1995–2005), and 1768 (2005–2016) manuscripts. The overall increase of about 250% indicates the strong interest in the field and that in the future we might witness a revolution in the study of protein–RNA networks!

Indeed, we do not have yet a complete understanding of how protein–RNA binding specificity is achieved and how the regulatory function of individual proteins is influenced by synergy and competition with other molecules. Novel approaches based on biochemical and functional studies, such as for instance SHAPE (Wan et al., 2011) and CRISPR (Hsu et al., 2014) paired with bioinformatics will lead to a better understanding of the principles underlying protein–RNA networks. In particular, advances based on high-resolution (STochastic Optical Reconstruction Microscopy, STORM) and biophysical (Nuclear Magnetic Resonance NMR) characterization will be key to derive mechanistic models for the interactions. Computational models will be an important source of information to identify new trends, understand the principles of molecular recognition and design experiments. Improvements in the theoretical models and validation of their predictions will be crucial to achieve a better description of the role of coding and non-coding RNAs in protein networks (Cirillo et al., 2014).

## Author contributions

GT wrote the “Grand Challenge” to introduce the most recent advances in the field of protein–RNA as well as the challenges that experimental and computational approaches will have to face in future studies.

## Funding

My research received funding from the European Union Seventh Framework Programme (FP7/2007-2013), through the European Research Council, under grant agreement RIBOMYLOME_309545 (GT), and from the Spanish Ministry of Economy and Competitiveness (BFU2014-55054-P). I also acknowledge support from AGAUR (2014 SGR 00685), the Spanish Ministry of Economy and Competitiveness, “Centro de Excelencia Severo Ochoa 2013–2017” (SEV-2012-0208).

### Conflict of interest statement

The author declares that the research was conducted in the absence of any commercial or financial relationships that could be construed as a potential conflict of interest.
